# Down-regulation of traditional oncomiRs in plasma of breast cancer patients

**DOI:** 10.18632/oncotarget.20484

**Published:** 2017-08-24

**Authors:** Dana Jurkovicova, Bozena Smolkova, Monika Magyerkova, Zuzana Sestakova, Viera Horvathova Kajabova, Ludovit Kulcsar, Iveta Zmetakova, Lenka Kalinkova, Tomas Krivulcik, Marian Karaba, Juraj Benca, Tatiana Sedlackova, Gabriel Minarik, Zuzana Cierna, Ludovit Danihel, Michal Mego, Miroslav Chovanec, Ivana Fridrichova

**Affiliations:** ^1^ KRD Molecular Technologies Ltd., Bratislava, Slovakia; ^2^ Department of Genetics, Cancer Research Institute, Biomedical Research Center, Slovak Academy of Sciences, Bratislava, Slovakia; ^3^ Department of Surgical Oncology, National Cancer Institute, Bratislava, Slovakia; ^4^ Medical Department of St. Elizabeth University, Bratislava, Slovakia; ^5^ Institute of Molecular Biomedicine, Faculty of Medicine, Comenius University, Bratislava, Slovakia; ^6^ Institute of Pathological Anatomy, Faculty of Medicine, Comenius University, University Hospital, Bratislava, Slovakia; ^7^ Pathological-Anatomical Workplace, Health Care Surveillance Authority, Bratislava, Slovakia; ^8^ 2^nd^ Department of Oncology, Faculty of Medicine, Comenius University, National Cancer Institute, Bratislava, Slovakia

**Keywords:** miR-17/92 cluster, miR-21, miR-27a, miR-155, down-regulation of oncomiRs

## Abstract

Deregulated expression of microRNAs has the oncogenic or tumor suppressor function in cancer. Since miRNAs in plasma are highly stable, their quantification could contribute to more precise cancer diagnosis, prognosis and therapy prediction. We have quantified expression of seven oncomiRs, namely miR-17/92 cluster (miR-17, miR-18a, miR-19a and miR-20a), miR-21, miR-27a and miR-155, in plasma of 137 breast cancer (BC) patients. We detected down-regulation of six miRNAs in patients with invasive BC compared to controls; however, only miR-20a and miR-27a down-regulations were statistically significant. Comparing miRNA expression between early and advanced stages of BC, we observed statistically significant decrease of miR-17 and miR-19a. We identified down-regulation of miR-17 and miR-20a in patients with clinical parameters of advanced BC (lymph node metastasis, tumor grade 3, circulating tumor cells, higher Ki-67-related proliferation, hormone receptor negativity and HER2 amplification), when compared to controls. Moreover, decreased level of miR-17 was found from low to high grade. Therefore, miR-17 could represent an indicator of advanced BC. Down-regulated miR-27a expression levels were observed in all clinical categories regardless of tumor progression. Hence, miR-27a could be used as a potential diagnostic marker for BC. Our data indicates that any changes in miRNA expression levels in BC patients in comparison to controls could be highly useful for cancer-associated pathology discrimination. Moreover, dynamics of miRNA expression changes could be used for BC progression monitoring.

## INTRODUCTION

Breast cancer (BC) is the most common female malignancy. In 2012, there have been almost 1.68 million new BC cases and 0.52 million BC deaths worldwide representing 25.2% and 14.7% of all cancers diagnosed in women, respectively [1]. Traditional classification based on clinico-pathological features, namely tumor size and grade, nodal involvement and immunohistochemical markers such as ER (estrogen receptor), PR (progesterone receptor), HER2 (erb-b2 receptor tyrosine kinase 2) and expression of Ki-67 proliferation marker, is the first step in patient management. Advances in molecular biology significantly helped to understand high heterogeneity of breast tumors and the first molecular classification subdivided breast tumors according to pattern of their gene expression [2]. In terms of biology, survival and recurrence rate, there are six intrinsic sub-groups generally accepted, namely Luminal A, Luminal B, Basal-like, Normal-like, HER2-enriched and Claudin-low [3–5].

Circulating tumor cells (CTCs) are cancer cells that can be identified in the blood of cancer patients and they play a role in the metastatic spread and the corresponding clinical consequences. Recently, strong prognostic value of CTCs in metastatic BC has been documented [6–7]. Moreover, the baseline CTC counts have been shown to be a useful early predictor of metastatic potential in BC patients [8].

Several years ago, a new class of small (approximately 22 nucleotides long) non-coding RNA molecules, microRNAs (miRNAs, miRs) was discovered [9]. miRNAs post-transcriptionally regulate gene expression of their targets and therefore they influence the majority of biological processes in a sequence-specific manner [10–11]. Interestingly, numerous studies showed a differential miRNA expression profile in cancer as compared to normal tissues, and a global miRNA down-regulation is considered to be a common feature of human malignancies [12–13]. miRNAs can act as tumor suppressors, oncogenes or metastasis-manipulators, thereby can actively participate in a variety of oncogenic processes [14–15]. miRNA expression profiles have been considered to be a promising tool for cancer diagnosis and more precise identification of BC sub-types and therefore can be a useful predictive and prognostic biomarkers [16– 17].

miR-17/92 cluster belongs to the most investigated cancer-related miRNA clusters. It is encoded by the *C13orf25* gene (also known as *MIR17HG*), firstly described in malignant lymphoma [18]. The members of this cluster regulate cell proliferation, cell cycle, apoptosis, angiogenesis, immune response and other essential biological processes in normal development, as well as in immune, cardiovascular and neurodegenerative diseases, aging and cancer [17, 19–21]. miR-17/92 cluster is located in the third intron of an approximately 7 kb primary transcript of *C13orf25.* It is a functional precursor of six individual miRNAs: miR-17, miR-18a, miR-19a, miR-20a, miR-19b-1 and miR-92a-1, which were found to be over-expressed in several types of cancer including BC [20, 22–23]. The increased levels of miR-17/92 in triple negative breast cancers (TNBC) compared to the other tumor sub-types have been reported previously [24]. miR-18a directly targets ER-alpha and this miRNA is highly expressed in ER-alpha-negative tumors as compared to ER-alpha-positive tumors, thus providing the first direct evidence of miRNAs inhibiting ER-alpha signaling in BC [25]. Targeted down-regulation of the *AIB1* gene (amplified in breast cancer 1) expression by miR-17-5p has been shown to result in decreased cell proliferation, indicating a possible tumor suppressor role of this miRNA in breast tumorigenesis. On the other hand, reduction or silencing of miR-17-5p expression led to an increase of the *AIB1* gene expression in 11 of 12 BC cell lines [26]. Moreover, reduced levels of miR-17 and miR-20a were shown in highly invasive BC cell lines and lymph node-positive BC in comparison to negative cases [27].

miR-21 is another deregulated miRNA involved in breast tumorigenesis. Qian and colleagues [28] found a variability in elevated miR-21 expression in 344 BC tissues and high miR-21 levels were associated with aggressive disease features in the early stage patients. Moreover, they documented positive correlation between high miR-21 and TGF-beta 1 (transforming growth factor beta 1) expression levels, suggesting that miR-21 levels are possibly up-regulated by TGF-beta 1 and might thus contribute to BC progression. Similarly, others showed over-expressed miR-21 level in 25 of 32 BC in comparison to matched normal breast tissues that correlated with presence of lymph node metastasis (LNM). Additionally, in four BC cell lines miR-21 levels inversely correlated with the expression of TIMP3 (TIMP metallopeptidase inhibitor 3), suppressing extracellular matrix degradation [29]. Importantly, increased miR-21 levels can distinguish normal breast tissue from ductal carcinoma *in situ* (DCIS) and invasive carcinomas [24]. Higher miR-21 expression was observed also in patients with more advanced disease requiring total mastectomy comparing to those after breast conserving surgery. Other associations of miR-21 over-expression with larger tumor size, higher stage and grade, ER negative and HER2 positive status, HER2 positive tumor sub-type, high Ki-67 and poor disease-free survival strongly suggest possible prognostic and predictive value of this miRNA in BC [30].

It has been documented that miR-27a may activate Wnt/β-catenin signaling pathway by negative regulation of SFRP1 (secreted frizzled related protein 1) affecting proliferation, migration and invasion of BC cells. This observation was supported by detection of higher miR-27a expression and lower SFRP1 mRNA and protein expression in BC when compared to normal breast tissues [31]. High miR-27a expression strongly correlated with the clinical stage and overall survival time of BC patients. Therefore, up-regulation of miR-27a might play an important role in disease progression. The oncogenic effect of miR-27a can be mediated through the regulation of the target *ZBTB10* (zinc finger and BTB domain containing 10) gene known to be involved in tumor growth, metastasis and chemotherapy resistance [32].

Traditional oncomiR, miR-155, has been found to be up-regulated in many cancers including BC. It has been shown that miR-155 performs its oncogenic role by targeting the *SOCS1* (suppressor of cytokine signaling 1) gene contributing to a constitutive STAT3 (signal transducer and activator of transcription 3) activation that suggests a potential bridging role of miR-155 between inflammation and tumorigenesis [33]. Moreover, miR-155 has been described as an independent prognostic factor for BC due to its higher levels in tumor tissues associated with advanced disease and worse survival [34].

In many studies, miRNAs have been described as free stable molecules in human plasma/serum that are protected from endogenous RNase activities by Argonaute-2 protein binding, high density of lipoproteins or by encapsulation in microvesicles or exosomes [35–38]. Compared to healthy individuals, the different levels of extracellular circulating miRNAs in plasma/serum were found in patients suffering from various diseases including cancer. Therefore, deregulated miRNA expression profiles can be utilized as a promising non-invasive biomarker for more efficient diagnosis and prognosis of BC.

Data on individual miRNA expression and deregulation in cancer patients are not uniform and in many cases they are rather controversial, especially if examined in body fluids, such as plasma or serum. In plasma samples of 152 BC patients, miR-17 and miR-155 were not deregulated in a tumor-specific manner, since their expression levels in patients without metastatic disease and controls were similar. However, the decreased levels of both miRNAs were observed in metastatic cases when compared to patients with non-metastatic cancers [39]. Others detected over-expression of miR-18a providing clear discrimination between plasma samples of cancer patients and healthy controls, but without any association between miRNA expression and tumor grade and size, menopausal or LNM status [40–41]. miR-21 was down-regulated in plasma specimens of TNBC in comparison to non-TNBC and healthy women and its levels were significantly increased before surgical removal of the tumor and reduced after the surgery [42–43]. Furthermore, significantly lower serum levels of miR-155 were observed in patients with metastatic than in non-metastatic BC. However, significantly higher miR-155 expression was detected in both sub-groups compared to healthy controls, indicating association of increased miR-155 plasma levels with tumor progression [44].

Taking into account a growing evidence of the role of miRNAs in cancer development and progression, we have investigated whether expression profiling of selected miRNAs could contribute to more precise classification of BC and discrimination of its advanced stage. In addition, a possibility of association between these miRNAs and the most important clinical parameters in BC has also been examined. Therefore, in the plasma samples of various BC patients we have quantified expression of seven oncomiRs, namely four members of miR-17/92 cluster (miR-17, miR-18a, miR-19a and miR-20a) and three other miRNAs (miR-21, miR-27a and miR-155) according to our previous experience [45]. We have found significantly down-regulated miR-20a and miR-27a in BC patients compared to healthy controls. Moreover, other two miRNAs, miR-17 and miR-19a, displayed substantial changes in their levels during process of breast tumorigenesis, specifically up-regulation in early and down-regulation in advanced stages of BC. Finally, miR-17 down-regulation has been associated with high tumor grade in contrast to miR-27a, which we observed to be down-regulated in all evaluated clinical categories regardless of tumor progression.

## RESULTS

### miRNA expression in plasma samples

**Figure 1:**
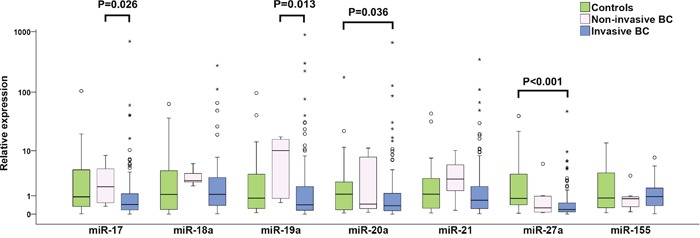
Individual expression levels of analyzed miRNAs in healthy controls, non-invasive and invasive BC patients The median is depicted by a horizontal line within each bar. The length of the boxes is the interquartile range (IQR) that represents values between the 75^th^ and 25^th^ percentiles of individual fold change values. Values more than 3 IQRs from the end of the box are labeled as extreme (*). Values more than 1.5 IQRs but less than 3 IQRs from the end of the box are labeled as outliers (O). The number of invasive BC, non-invasive BC and controls included in the study were 128, 9 and 28, respectively. For each miRNA, only samples analyzed successfully were included into statistical analysis. Abbreviation: BC, breast cancer.

In the group of 128 patients with invasive BC (Table 1), the relative expression levels of six studied miRNAs, miR-17, miR-19a, miR-20a, miR-21, miR-27a and miR-155, were down-regulated in comparison to healthy controls and the corresponding fold changes were 0.49, 0.57, 0.34, 0.70, 0.21 and 0.71, respectively (Table 2). However, only down-regulations of miR-20a (P=0.036) and miR-27a (P<0.001) were statistically significant (Figure 1). In individual patients with invasive BC, both up-regulation and down-regulation of the studied oncomiRs was recorded, with the latter being prevailing event: 70.6, 64.5, 76.8, 60.7, 81.4 and 53.4% of these patients manifested down-regulation of miR-17, miR-19a, miR-20a, miR-21, miR-27a and miR-155 expression, respectively. On the other hand, miR-18a was up-regulated in 54.4% of BC patients.

**Table 1:** Clinico-pathological characteristics of patients with invasive BC

Variables	N	%
**All**	128	100.0
**Age (years)**		
**≤50**	35	27.3
**>50**	93	72.7
**Tumour size (mm)**		
**≤ 20**	86	67.2
**> 20**	42	32.8
**LNM status^a^**		
**0**	85	66.9
**>1**	42	33.1
**Grade**		
**G1 and G2**	81	64.3
**G3**	45	35.7
**Tumor histology**		
**DIC**	115	90.6
**Others^b^**	12	9.4
**Hormone receptor status^c^**		
**Negative**	23	18.1
**Positive**	104	81.9
**HER2 status**		
**Normal**	106	82.8
**Amplified**	22	17.2
**Ki-67^d^**		
**Low**	80	62.5
**High**	48	37.5
**CTC presence^e^**		
**Negative**	92	74.2
**Positive**	32	25.8

^a^ lymph node metastasis status was categorized according to the number of metastatic lymph nodes

^b^ lobular invasive carcinomas, invasive ductal carcinomas with tubular features, mucinous and mixed mucinous carcinomas

^c^ negative for both (estrogen and progesterone receptor) or positive for either with cut-off 10%

^d^ cut-off 20%

^e^ CTC were detected through quantification of EMT-inducing transcription factor gene transcripts and epithelial antigen

Abbreviations: LMN, lymph node metastasis; G1, G2 and G3, Grade 1, 2 and 3, respectively; DIC, ductal invasive carcinoma; HER2, erb-b2 receptor tyrosine kinase 2; Ki-67, Ki-67 proliferation marker; CTC, circulating tumor cells.

**Table 2:** Comparison of miRNA expression among healthy controls, non-invasive and invasive BC patients

	Non-invasive BC *vs.* controls		Invasive BC *vs.* controls		Invasive *vs.* non-invasive BC	
	Fold change	P value	Fold change	P value	Fold change	P value
**miR-17**	1.64	0.534	0.49	0.096	0.30	**0.026**
**miR-18a**	2.98	0.352	1.09	0.850	0.37	0.209
**miR-19a**	3.95	0.097	0.57	0.229	0.14	**0.013**
**miR-20a**	0.80	0.914	0.34	**0.036**	0.43	0.278
**miR-21**	1.88	0.243	0.70	0.358	0.37	0.093
**miR-27a**	0.32	0.114	0.21	**<0.001**	0.68	0.515
**miR-155**	0.58	0.384	0.71	0.301	1.21	0.378

Notes: P values shown in bold indicate statistical significance of the expression level. Analysis of statistical significance was applied to the ΔCt (threshold cycle) values. The numbers of invasive BC, non-invasive BC and controls included in the study were 128, 9 and 28, respectively. For each miRNA, only samples analyzed successfully were included into statistical analysis.

BC, breast cancer.

**Figure 2:**
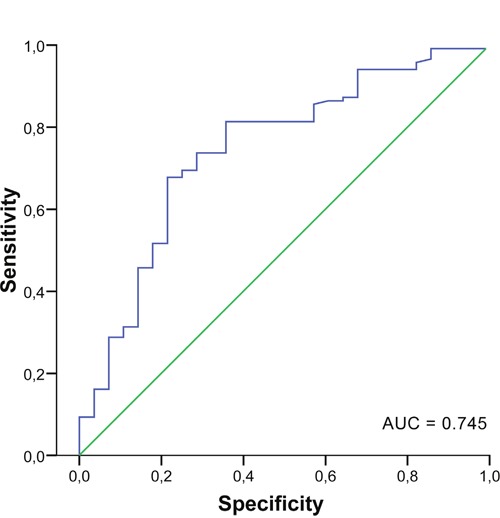
Receiver operator characteristic curve for miR-27a AUC, area under the curve.

To evaluate diagnostic potential of analyzed miRNAs, multivariate analysis was used. The results of binary logistic regression adjusted for age indicate that three miRNAs, miR-18a, miR-21 and mir-27a, are highly relevant in this manner. miR-27a displays the highest statistical significance to distinguish invasive BC patients from healthy controls (P<001; odds ratio = 0.22, 95% CI = 0.104-0.475) (Table 3). The classification performance of these miRNAs was addressed using discriminant analysis. As shown in Table 4, 87.9% of original grouped cases were correctly classified based on the miR-18a, miR-21 and mir-27a expression profiles. Receiver operating characteristic (ROC) curve analyses were applied to obtain diagnostic utility of the differentially expressed miRNAs. Area under curve (AUC) for miR-27a was 0.745 (95% CI = 0.640-0.849; p < 0.001) (Figure 2), while non-significant results were found for miR-18a and miR-21 with AUC values 0.504 and 0.559, respectively.

**Table 3:** Binary logistic regression (adjusted for age) for the relationship between analyzed miRNAs and invasive BC

Variable	Coefficient	Standard error	Wald	P value	Odds ratio	95% CI
**miR-18a**	0.484	0.180	7.205	0.007	1.623	1.140-2.312
**miR-21**	0.766	0.260	8.698	0.003	2.151	1.293-3.579
**miR-27a**	-1.503	0.387	15.086	<0.001	0.222	0.104-0.475

The number of invasive BC included in the study was 128. For each miRNA, only samples analyzed successfully were included into statistical analysis.

BC, breast cancer.

**Table 4:** Discriminant analysis results for miR-18a, miR-21 and miR-27a expression profiles

Original group	Predicted group membership		Total
	Controls N (%)	Invasive BC N (%)	
**Controls N (%)**	13 (59.1)	9 (40.9)	22 (100.0)
**Invasive BC N (%)**	4 (4.7)	81 (95.3)	85 (100.0)

87.9% of original grouped cases correctly classified. For each miRNA, only samples analyzed successfully were included into statistical analysis.

BC, breast cancer.

### miRNA expression in plasma of invasive BC *versus* clinico-histopathological characteristics

A relationship between miRNA expressions and clinico-histopathological characteristics, namely tumor size, grade and histology, regional lymph node involvement, HR (estrogen and progesterone) and HER2 status, Ki-67 expression and CTC presence, was evaluated in 128 patients suffering from invasive BC. Numerous associations between evaluated miRNAs and all clinico-histopathological categories except tumor size were observed (Table 5). Statistically significant differences in the miR-19a and miR-20a expression levels in LNM positive BC in comparison to controls were recorded (P=0.032 and 0.024, respectively), with both miRNAs being down-regulated. Moreover, significantly different miR-19a expression level between LNM negative and positive BC was observed (P=0.046). Regarding tumor grade 3 (G3), significantly lower levels of miR-17 and miR-20a expression were detected (P=0.009 and 0.025, respectively) in comparison to controls. In addition, differences between G1 and G2 *versus* G3 in the miR-17 and miR-18a expression levels were found (P= 0.007 and 0.045, respectively). Regarding HR status, significant difference in the miR-17 expression level was found between HR negative cases and controls (P=0.010), as well as between HR positive and negative patients (P=0.023). miR-17 was also significantly down-regulated in the samples with amplified levels of HER2 compared to healthy controls (P=0.046). Interestingly, miR-20a expression was considerably down-regulated in the patient's samples with either normal or amplified levels of HER2 as compared to controls (both P=0.033). In cases with high proliferation in tumors represented by high Ki-67 expression, significant down-regulation of miR-17 and miR-20a expressions was also observed as compared to controls (P=0.017 and 0.016). In patients with CTC in peripheral blood, significantly down-regulated miR-20a expression was detected in comparison to controls (P=0.023). HR positivity (HR positive *versus* negative) and high levels of Ki-67 (Ki-67 high *versus* low) were associated with up-regulation of miR-155 (P=0.022 and P=0.039, respectively). In all six clinico-histopathological categories, the expression of miR-27a was significantly down-regulated in groups matched to healthy controls (for all P≤0.001).

**Table 5:** Comparison of miRNA expression levels between healthy controls and invasive BC patients (stratified by clinical characteristics)

	Fold change	P value	Fold change	P value	Fold change	P value
LNM	LNM negative *vs*. Controls		LNM positive *vs*. Controls		LNM positive *vs*. LNM negative	
**miR-19a**	0.75	0.552	0.32	**0.032**	0.42	**0.046**
**miR-20a**	0.38	0.073	0.27	**0.024**	0.72	0.458
**miR-27a**	0.23	**<0.001**	0.19	**<0.001**	0.84	0.557
**G (grade)**	**G1 and G2** ***vs*. Controls**		**G3** ***vs*. Controls**		**G3** ***vs*. G1 and G2**	
**miR-17**	0.77	0.364	0.28	**0.009**	0.36	**0.007**
**miR-18a**	1.47	0.445	0.67	0.494	0.46	**0.045**
**miR-20a**	0.43	0.110	0.26	**0.025**	0.61	0.136
**miR-27a**	0.26	**0.001**	0.17	**<0.001**	0.66	0.674
**HR status**	**HR positive** ***vs.*** **Controls**		**HR negative** ***vs*. Controls**		**HR positive** ***vs*. HR negative**	
**miR-17**	0.61	0.159	0.21	**0.010**	0.35	**0.023**
**miR-20a**	0.37	**0.042**	0.29	0.060	0.78	0.817
**miR-27a**	0.23	**<0.001**	0.14	**<0.001**	0.58	0.303
**miR-155**	0.62	0.181	1.42	0.324	2.34	**0.022**
**HER2 status**	**HER2 normal** ***vs*. Controls**		**HER2 amplified** ***vs*. Controls**		**HER2 amplified** ***vs*. HER2 normal**	
**miR-17**	0.54	0.143	0.30	**0.046**	0.55	0.085
**miR-20a**	0.37	**0.033**	0.24	**0.033**	0.63	0.265
**miR-27a**	0.24	**<0.001**	0.12	**<0.001**	0.50	0.238
**Ki-67 expression**	**Ki-67 low** ***vs.*** **Controls**		**Ki-67 high** ***vs.*** **Controls**		**Ki-67 high** ***vs.*** **Ki-67 low**	
**miR-17**	0.62	0.315	0.32	**0.017**	0.52	0.080
**miR-20a**	0.42	0.105	0.25	**0.016**	0.60	0.159
**miR-27a**	0.22	**<0.001**	0.20	**<0.001**	0.89	0.673
**miR-155**	0.58	0.247	1.01	0.786	1.74	**0.039**
**CTC positivity**	**CTC negative** ***vs.*** **Controls**		**CTC positive** ***vs.*** **Controls**		**CTC positive** ***vs.*** **CTC negative**	
**miR-20a**	0.42	0.112	0.26	**0.023**	0.62	0.137
**miR-27a**	0.23	**<0.001**	0.20	**0.001**	0.88	0.695

Only miRNAs significantly up- or down-regulated are listed. P values shown in bold indicate statistical significance of the expression level. Analysis of statistical significance was applied to the ΔCt values. The numbers of invasive BC patients and controls included in the study were 128 and 28, respectively. For each miRNA, only samples analyzed successfully were included into statistical analysis.

LMN, lymph node metastasis; G1, G2 and G3, Grade 1, 2 and 3, respectively; HR, hormonal (estrogen and/or progesterone) receptor; HER2, erb-b2 receptor tyrosine kinase 2; Ki-67, Ki-67 proliferation marker; CTC, circulating tumor cells.

The discrimination analysis was used to test classification performance of analyzed miRNAs for disease course, namely the presence of LNM, G3, HR negativity, HER2 amplification, high Ki67-proliferation and CTC positivity. Significant results were found for LNM status and tumor grade; however, as shown in Table 6, only 60.8% of original group cases were correctly classified for LNM status and 62.6% for grade based on the miR-17 and miR-19a expression profiles, respectively.

**Table 6:** Discriminant analysis results for clinical characteristics

Original group	Predicted group membership		
	**LNM negative N (%)**	**LNM positive N (%)**	**Total**
**LNM negative N (%)**	62 (78.5)	17 (21.5)	79 (100.0)
**LNM positive N (%)**	30 (73.2)	11 (26.8)	41 (100.0)
	**G1 and G2 N (%)**	**G3 N (%)**	**Total**
**G1 and G2 N (%)**	48 (72.7)	18 (27.3)	66 (100.0)
**G 3 N (%)**	22 (53.7)	19 (46.3)	41 (100.0)

60.8% of original grouped cases correctly classified for LNM, 62.6% of original grouped cases correctly classified for tumor grade. For each miRNA, only samples analyzed successfully were included into statistical analysis.

LMN, lymph node metastasis; G1, G2 and G3, Grade 1, 2 and 3, respectively.

### miRNA expression changes in plasma in different stages of tumorigenesis

**Figure 3:**
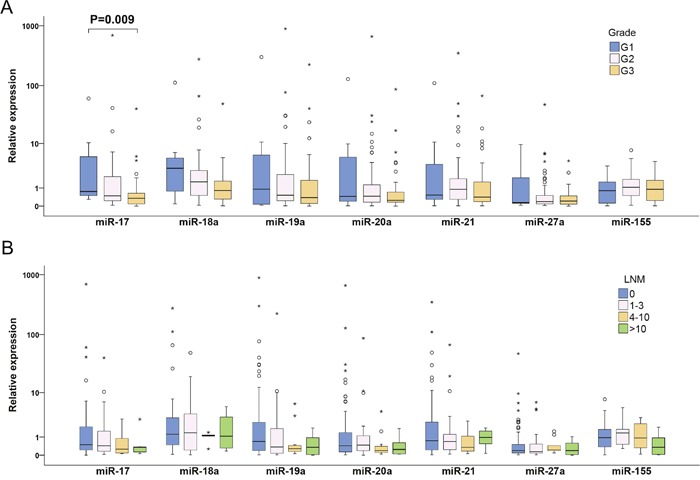
Individual expression levels of analyzed miRNAs in different sub-groups of invasive BC patients classified according to tumor grade (A) and LNM status (B) The median is depicted by a horizontal line within each bar. The length of the boxes is the interquartile range (IQR) that represents values between the 75^th^ and 25^th^ percentiles of individual fold change values. Values more than three IQRs from the end of the box are labeled as extreme (*). Values more than 1.5 IQRs but less than 3 IQRs from the end of the box are labeled as outliers (O). The number of invasive BC was 128. For each miRNA, only samples analyzed successfully were included into statistical analysis. Abbreviations: G1, G2 and G3, Grade 1, 2 and 3, respectively; LNM, lymph node metastasis; BC, breast cancer.

To examine changes in miRNA expression during breast tumorigenesis, miRNA expression in plasma of patients with nine non-invasive and 128 invasive BC were analyzed. In the case of miR-17 and miR-19a, increased and decreased expressions in patients with non-invasive and invasive BC were observed, respectively. Moreover, significant differences between these two groups were found (P=0.026 and P=0.013) (Table 2, Figure 1). miR-20a and miR-27a were down-regulated in patients with invasive BC compared to controls (P= 0.036 and P<0.001) (Table 2, Figure 1). In patients with invasive tumors, more detailed analysis of advanced carcinoma characteristics in LNM (0, 1-3, 4-10 and >10 of metastatic lymph nodes) and tumor grade (G1, G2 and G3) sub-groups documented that miR-17 and miR-19a expression levels decreased with increasing number of LNMs and that reduction of miR-17, miR-18a and miR-19a expression levels was observed from low to high grade. However, the only significant difference was found for miR-17 expression between G1 and G3 sub-groups (P=0.009) (Figure 3).

### miRNA expression *versus* protein expression of the target genes

**Figure 4:**
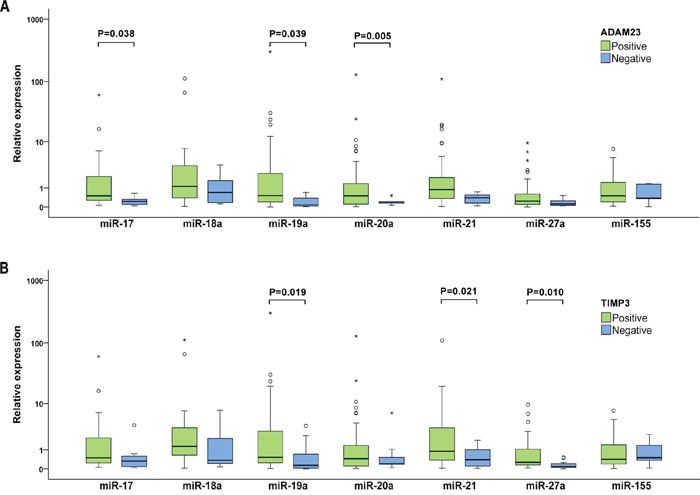
Individual expression levels of analyzed miRNAs stratified by the ADAM23 (A) and TIMP3 (B) protein expression The median is depicted by a horizontal line within each bar. The length of the boxes is the interquartile range (IQR) that represents values between the 75^th^ and 25^th^ percentiles of individual fold change values. Values more than 3 interquartile ranges (IQRs) from the end of the box are labeled as extreme (*). Values more than 1.5 IQRs but less than 3 IQRs from the end of the box are labeled as outliers (O). Data on protein expression were available for 55 invasive BC patients. For each miRNA, only samples analyzed successfully were included into statistical analysis.

To investigate the potential targets of the studied miRNAs in BC patients, the relationship between miRNA expression levels in plasma samples and expression of nine selected cancer associated proteins in tumor tissues was analyzed. Of them, the significant association between deregulated expression of five miRNAs (miR-17, miR-19a, miR-20a, miR -21 and miR -27a) and expression of two proteins contributing to invasion, angiogenesis and metastases regulation, transmembrane metalloproteinase-disintegrin ADAM23 and tissue inhibitor metalloprotease TIMP3, was observed (Figure 4). In patients without ADAM23 protein expression in tumors, miR-17, miR-19a and miR-20a were significantly down-regulated (P=0.038, 0.039 and 0.005) and the corresponding fold changes were 0.181, 0.1 and 0.136, respectively. Similarly, miR-19a, miR-21 and miR-27a were remarkably down-regulated in BC patients with inhibited TIMP3 protein expression (P=0.019, 0.021 and 0.01) with fold changes being 0.151, 0.292 and 0.075, respectively. The ADAM23 protein encoding gene has not been reported to be a target gene of any evaluated miRNA yet and our results also do not indicate any association. As mentioned previously, the encoding gene for the TIMP3 protein has been experimentally validated as target of miR-17 and miR-21. However, in our group of BC patients the possible direct targeting of the *TIMP3* gene by miR-17 and miR-21 *via* decreasing of the TIMP3 protein expression was not confirmed.

## DISCUSSION

Recently, the aberrant miRNA profiles in human tumors and liquid biopsies from cancer patients have been found, indicating their potential for the clinical use as diagnostic, predictive, or prognostic biomarkers. While human oncomiRs are usually highly expressed in cancer patients and contribute to tumor progression, miRsupps (oncosupressormiRs) inhibit tumorigenesis and are frequently degraded in various cancers [13, 15].

In our study, we have investigated four members of miRNA cluster with well-known oncogenic potential, miR-17/92 [22]. We found significantly decreased levels of miR-20a in patients with invasive BC compared to healthy controls and lower levels of miR-17 and miR-19a in invasive than in non-invasive BC. It has been documented that miRNAs located within miR-17/92 cluster are integrated in many essential biological process. Hence, any changes of their expression can considerably imbalance normal physiological conditions leading to pathological events. Expression of this cluster is controlled by multiple upstream regulators that either inhibit miR-17/92 expression through p53 or activate transcription of this cluster *via* c-Myc proto-oncogene and Aurora kinase A. Some of c-Myc transcription targets are in turn regulated by miR-17/92 that guarantees a delicate balance between miRNAs and relevant mRNAs in normal cells. Disruption of this equilibrium could contribute to aging and development of many diseases including cancer [15, 20, 46]. The oncogenic effect of miR-17/92 cluster is enhanced by cooperation among its individual members in targeting of tumor suppressors, as well as pathways such as PTEN and TGF-beta signaling [47]. It has been shown previously that in tumor tissues, the individual miRNAs of miR-17/92 cluster are mostly up-regulated [12, 20], although the results of several studies indicate the anti-invasive effects of miR-17, miR-19a and miR-20a. Down-regulation of miR-17, miR-17/20 and miR-19a in cell lines is associated with higher expression of the *AIB1, CXCL8* and *CCND* genes, as well as the *FRA1* proto-oncogene, resulting in an enhancement of cell proliferation, migration and metastasis [26–27, 48]. miR-17 metastatic suppression role was supported by Fan and colleagues [49], who documented that miR-17 inhibition triggers expression of multiple pro-metastatic genes in BC cells. In addition, it adopts cells to gain the metastatic features and leads to development of lung metastases. Importantly, after intratumoral administration of miR-17 reduction of lung metastasis was observed [49]. We hypothesize that down-regulation of miR-17, miR-19a and miR-20a in plasma samples of the BC patients in our study could indicate expression of pro-metastatic genes in tumor tissues that contribute to cancer progression. Interestingly, the post-transcriptional level of individual members of miR-17/92 cluster remarkably differs regardless of the joint transcription of the primary miRNA transcripts. This could be caused by different degree of protection or degradation of single miRNA members after the pre-miRNA status, as found for miR-18 [21, 50]. We have also observed this phenomenon in plasma samples of BC patients who manifested down-regulation of miR-17, miR-19a and miR-20a, but up-regulation of miR-18a expression, indicating a possibility of selective protection against degradation in the case of miR-18a.

The results of our and previous studies have shown that miRNA expression profiles are modified during the process of tumorigenesis. Comparing the expression of studied miRNAs, we observed obvious increasing and decreasing levels in patients with non-invasive and invasive BC, respectively, as well as statistical difference between *in situ* and invasive BCs for both miR-17 and miR-19a. Similarly, different expression of miR-21 in tissues from atypical ductal hyperplasia, DCIS and DIC compared to normal breast was previously shown; however, with the increasing values during the tumor progression [51], that is in contrast to a decreasing trend in plasma from patients with non-invasive to patients with invasive BC in our study.

To investigate the association between clinico-histopathological features and miRNA expression levels, we found interesting links between features of carcinomas with worse prognosis such as LNM presence, G3, HR negative and HER2 amplified status, high Ki-67 expression and CTC presence, and down-regulation of the selected miRNAs of miR-17/92 cluster. We observed lower miR-17 expression in plasma from patients with G3, HR negativity, HER2 amplification and higher Ki-67 and decreased miR-19a expression associated with LNM. Furthermore, down-regulation of miR-20a was found in plasma of patients displaying all evaluated unfavorable clinical characteristics. These results indicate that decreased miR-17 and miR-20a expression levels could indicate tumor progression, but by using multivariate analysis we confirmed only association of miR-17 down-regulation with high tumor grade. In other studies, miRNA expression in plasma of patients without and with distal metastasis was evaluated and decreased miR-17 and miR-155 expression levels were reported to be associated with metastatic disease [39, 44]. In contrast, our data on tumor progression did not show significantly lower miR-17 and miR-155 expression levels in LNM positive compared to LNM negative samples. On the other hand, we found down-regulated miR-27a in all evaluated categories regardless of tumor progression. Therefore, miR-27a could be used as a potential diagnostic marker for BC. In line with the assumption of miRNA-27a being a marker for BC, others defined miR-27a as a prognostic marker for BC progression and patient survival [32].

In our previous study, we did not find any association between inhibition of the cancer-associated APC, ADAM23, CXCL12, E-cadherin, RASSF1, SYK, TIMP3, BRMS1 and SOCS1 proteins and promoter methylation of the encoding genes in BC patients [52]. Therefore, these proteins could be inactivated through another epigenetic mechanism, such as *via* targeting of the corresponding mRNA by miRNA. Moreover, experimentally validated data from public miRTarbase documents that *APC*, *TIMP3* and *SOCS1* are indeed the genes targeted by miR-27a and miR-155, miR-17 and miR-21, and miR-19a and miR-155, respectively [53]. In our group of patients without the ADAM23 and TIMP3 protein expression in tumors, we found down-regulated miR-17, miR-19a and miR-20a, and miR-19a, miR-21 and miR-27a levels, respectively. Inactivation of both proteins plays key role in processes of malignant transformation. Generally, metalloproteases induce tumor growth or survival, invasion, angiogenesis and metastasis, but metalloproteinase-disintegrins (ADAMs) are dedicated to regulation of growth signaling and adhesion of tumor cells. On the other hand, tissue inhibitors of metalloproteases (TIMPs) selectively inactivate metalloproteases that influence apoptosis and structure of extracellular matrix [54]. Regardless of the fact that our results did not indicate the direct miRNA targeting of the *ADAM23* and *TIMP3* genes, it is still possible that another type of interaction could be involved. The indirect mechanisms of mRNA transcription regulation was shown in several cell lines, in which the specific miRNA attached to the AU-rich region of target mRNA recruits the repressor protein complexes, thus inhibiting the gene expression [55, 56]. Another known possibility is direct binding of specific miRNA to the 5'UTR of target mRNA that induces transcription of the target gene, as shown in human fetal kidney and Wilms’ tumors [57]. Based on our knowledge, the only association between the TIMP3 protein and miR-21 expression was found in BC and in contrast with our results, miR-21 being up-regulated predominantly in LNM positive patients [29].

miRNAs in plasma/serum samples are very stable [35], therefore their quantification and profiling could represent a useful tool for more precise diagnosis, prognosis and prediction of therapy. A positive correlation between breast tumor tissue and sera in terms of the expression level of six miRNAs including miR-21 and miR-155 has been shown previously [58]. Using a high-throughput sequencing, significant differences in the levels of a number of aberrantly expressed miRNAs have been shown between BC tissue and serum. Nevertheless, eight miRNAs (including miR-18a, miR-19a and miR-20a) were consistently down-regulated and one up-regulated in both tumor and serum samples [59]. Other authors showed profiles of eight selected miRNAs concordantly up-regulated and one miRNA concordantly down-regulated in both plasma and tumor tissue of BC patients, but the levels of only three miRNAs out of eight were significantly increased before and decreased after surgery [43]. Other study investigated whether miRNAs in plasma are directly derived from tumor tissues, and whether longitudinal monitoring of miRNA expression profiles could provide the manifestation of disease recurrence. The authors did not find significant differences in miRNA expression levels during the longitudinal observation after surgery and therefore the circulating populations could be derived from tumor only partially. They recommended being careful in utility of plasma samples in cancer recurrence monitoring [60]. Overall, one of the causes of variability in data found in the literature could be different normalizers and different normalizing methods that are used for miRNA expression evaluation and comparison in plasma samples. Recent articles presented that the major contributors to circulating miRNA are blood cells and miRNA levels in plasma can be influenced by changes in blood cell counts and hemolysis. Hence, the interpretation of miRNA measurements may reflect more blood cell derived background than cancer specific origin [61]. Differences in miRNA profiles between plasma and tumor tissues could be related to miRNAs from blood cells that are also important components of tumor microenvironment. It is also possible that blood cells could influence the particular events in cancer progression *via* intracellular transfer of its miRNAs to various cells of microenvironment using experimentally confirmed mechanism [38].

A growing number of available biological datasets allowed development of computational methods to uncover the potential associations between miRNAs and various diseases. Development of these methods is based on the assumption that structurally and/or functionally similar miRNAs tend to play roles in similar biological (including pathological) processes and *vice versa*. The main advantage of applying computational methods to identifying miRNA-disease associations is their ability to pick up most promising miRNAs for further analysis with limited need for experimental methods that are demanding, time-consuming and costly. BC was one of the subjects for which the performance of computational methods, namely RWRMDA (Random Walk with Restart for MiRNA-Disease Association) was evaluated [62]. Importantly, 98% of the top 50 potential miRNAs of BC predicted by this method were confirmed to be true, indicating that computational methods can recover known experimentally verified miRNA-disease associations and hence has the potential to uncover potential miRNA-disease associations. Of our selected miRNAs, miR-27a was picked up by RWRMDA further strengthening its use as a potential diagnostic marker for BC.

During normal and pathological development of human organism, the dynamic changes in miRNA expression profiles are performed in time and tissue specific manner. The growing list of directly or indirectly targeted genes by individual miRNAs is still in progress and therefore it is difficult to predict how the aberrant miRNA profiles can influence the regulation of various biological processes and multiple signaling pathways. In our study, we present association between several important features of advanced BC and the altered levels of miR-17, miR-19a and miR-20a. Moreover, we show a universal cancer specific miR-27a down-regulation in plasma samples of BC patients. Our results support the concept that regardless of many discrepancies in miRNA profiles in different biological materials, the measurements of specific combination of miRNAs represent the promising diagnostic and prognostic tool.

## PATIENTS AND METHODS

### Patient samples

Out of 137 plasma samples of non-familial BC patients, 128 patients suffered from invasive tumors (ductal - DIC or others as lobular - LIC, invasive ductal carcinomas with tubular features, mucinous and mixed mucinous carcinomas) and nine from DCIS or LCIS (lobular carcinomas *in situ*). Samples were collected between March 2012 and March 2014 at the National Cancer Institute in Bratislava. Control plasma samples from 28 healthy women were collected in previous studies. The study was approved by the Institutional Review Board of the National Cancer Institute of Slovakia, and written informed consent was obtained from all patients and controls. The relevant clinical and histopathological data were retrieved from the patients’ clinical records, and tumors were characterized according to the TNM classification. At the time of BC diagnosis, the age of patients with invasive and non-invasive tumors ranged from 24 to 83 and 37 to 67 years (mean, 58.7± 11.7 and 51.0±9.8 years), respectively. Typing was performed according to the current WHO classification for breast neoplasms. No preoperative radiotherapy or chemotherapy had been performed in any of the cases before sample collection. In all patients with invasive tumors, the clinico-pathological data including age, tumor size, grade and histology, as well as regional lymph node involvement, HR (estrogen and progesterone) and HER2 status, Ki-67 expression and CTC presence were recorded (Table 1). In the group of nine patients with non-invasive cancers, eight DCIS, one LCIS, two ER negative and four HER2 positive cases were included. Controls were at the age from 20 to 78 with the mean 53.6. *±* 15.6 and they had no signs and symptoms of cancer or other serious diseases. No statistical differences in mean age between patients with invasive and non-invasive cancers and controls were found by performing the pairwise comparison tests.

### Plasma preparation of RNA isolation

For plasma collection, 5 ml of peripheral blood were withdrawn from patients and healthy controls. Samples were kept at room temperature and processed within one hour. Separation of plasma was accomplished by centrifugation at 2500 rpm for 10 min at room temperature and 3500 rpm for 10 min afterwards to remove the cell debris. Plasma was recovered and stored at -80°C until analyzed.

For qRT-PCR analysis, total RNA was extracted from 300 μl of sera using TRI Reagent®BD for processing whole blood, plasma, or serum (Life technologies, USA). Total RNA was quantified using the NanoDrop ND-1000 Spectrophotometer (Thermo Scientific, USA) and the Qubit fluorometer (Qubit® RNA HS Assay Kit, LifeTechnologies, USA).

### qRT-PCR quantification of miRNA

Expression of oncogenic miRNAs (miR-17, miR-18a, miR-19a, miR-20a, miR-21, miR-27a and miR-155) was evaluated by qRT-PCR. Expression of mature miRNAs was determined using First-Strand cDNA Synthesis System (Central European Biosystems, Czech Republic) supplemented with poly(A)polymerase (Takara, Japan) and ATP (Sigma, Germany). Briefly, for cDNA synthesis 100 ng of total RNA in a final volume of 10 μl including 1μl of 10x poly(A)polymerase buffer, 0.1 mM of ATP, 1μM of RT-primer, 0.1 mM of each deoxynucleotide (dATP, dCTP, dGTP and dTTP), 100 units of MuLV (Murine Leukemia Virus) reverse transcriptase and 1 unit of poly(A)polymerase were incubated at 42°C for 1 hour followed by enzyme inactivation at 95°C for 5 minutes. The sequence of the oligo-d(T)/adapter primer was 5’-CAGGTCCAGTTTTTTTTTTTTTTTVN, where V is A, C and G and N is A, C, G and T (IDT, Leuven, Belgium).

In each sample, cDNA yield was quantified using the NanoDrop ND-1000 Spectrophotometer (Thermo Scientific, USA) and the Qubit fluorometer (Qubit® RNA HS Assay Kit, Life Technologies, USA) to use equal amounts of cDNA (300 ng in 1 μl) for real-time PCR.

Real-time PCR detection and quantification of mature forms of hsa-miR-17-5p, hsa-miR-18a-5p, hsa-miR-19a-3p, hsa-miR-20a-5p, hsa-miR-21-5p, hsa-miR-27a-3p, hsa-miR-155-5p were performed using SYBR Premix Ex Taq II (Tli RNaseH Plus), ROX plus (Takara, Japan), adapter-specific reverse primer and miRNA-specific forward primers ( http://registre.indprop.gov.sk, Utility models: 6932, UV7154, 7155, 7157-7159 and for miR-19a-3p: 5` CAGTGTGCAAATCTATGCAA` 3).

qPCR was performed using BIOER, LineGene 9660 Real-Time PCR System (Hangzhou Bioer Technology Co., Ltd, China) at following settings: 95°C for 5 min, followed by 40 cycles at 95°C for 20 sec and 60°C for 50 sec, followed by melt cycle. Each amplification of mature miRNA was done in triplicate. Ct values were normalized against reference control Snord44. After testing expression of several reference control genes, Snord44 was selected due to its most stable expression in both BC patient samples and healthy controls. In patient samples, the mean±SD of Ct defined as a number of cycles required for the fluorescent signal to cross the threshold, was 27.08±1.14 and in healthy controls 27.62±1.98.

### CTC detection

Detailed description of the original CTC detection method was published recently by Cierna and colleagues [63]. Briefly, the protocol consists of three major steps: CD45^+^ cell depletion, RNA extraction, and identification of EMT-inducing factors and epithelial gene transcripts in CD45-enriched subsets. RNA was analyzed for expression of EMT-inducing transcription factor gene transcripts (TWIST1, SNAIL1, SLUG, and ZEB1) and epithelial antigen (CK19) by RT-PCRs using TaqMan probes. Expressions of the genes of interest were calibrated using the housekeeping gene *GAPDH* and Δ-Ct method was used for quantification of the target genes. The values of healthy donors were used as cutoff to determine CTC positivity.

### Immunohistochemistry

For 55 invasive BC cases the results of immunohistochemical (IHC) analyses of 9 proteins associated with cell growth regulation, cell adhesion, invasiveness, and metastasis (APC, ADAM23, CXCL12, E-cadherin, RASSF1, SYK, TIMP3, BRMS1 and SOCS1) were available from previous study [52]. In this set, three proteins encoded by experimentally validated targeting genes of evaluated miRNAs were included, namely the *APC* gene is targeted by miR-27a and miR-155, the *TIMP3* gene is targeted by miR-17 and miR-21 and the *SOCS1* gene is targeted by miR-19a and miR-155 [53]. Protein expressions were detected *via* semi-quantitative IHC analyses of tissue microarrays using ImmunoReactive Score (IRS), as described previously [64]. The scores from staining intensity and positive cells were multiplied, giving quotients ranging between 0 and 12; protein expression was stratified as negative (0) or positive with a staining intensity between 1 and 12.

### Statistical analysis

SPSS 23.0 was used for statistical analysis of the data. Normality of distribution was tested by Kolmogorov-Smirnoff and Shapiro-Wilk tests. Relative quantification of miRNA expression was calculated with 2^-ΔΔCt^ method, which represents relative fold changes of miRNA expression. Therefore, ΔΔCt = ΔCt (tumor group or unfavorable clinical condition) − ΔCt (control group or more favorable clinical condition).

Analysis of the significance of fold change in miRNA expression between studied groups was applied to the ΔCt values. If normally distributed, data were tested by Student's t-test or analysis of variance (ANOVA) with Bonferroni's or Tamhane's tests for multiple comparisons. The nonparametric Mann-Whitney U test was used for non-normally distributed data. All tests were two-tailed, performed at the significance level α = 0.05. Categorical data were tested by Chi square testing. Binary logistic regression model was used to calculate odds ratio with 95% confidence interval to estimate association and control for potential confounding factors. Discriminant analysis was performed to determine discriminative ability of the screened plasma miRNAs. The receiver-operator characteristic (ROC) analyses were applied to evaluate the diagnostic and predictive accuracy of significantly associated miRNAs.
